# Transcription Factor NKX6.3 Sheds Light on Gastric Cancer Progression

**DOI:** 10.1016/j.ebiom.2016.06.021

**Published:** 2016-06-16

**Authors:** Alison C. Testa, Alistair R.R. Forrest

**Affiliations:** Harry Perkins Institute of Medical Research, QEII Medical Centre and Centre for Medical Research, The University of Western Australia, Nedlands, Western Australia, Australia

The Nkx factors are a family of homeodomain containing transcription factors known to have tissue specific expression and thought to play a regulatory role in organ development. Certain members of this family have been studied in detail and found to play a role in disease. For example, NKX6-1 is involved in pancreatic development and serves as a marker for pancreatic and duodenal neuroendocrine tumors (Tseng et al., 2015). Evidence also supports the role of NKX2-1 as an oncogene in lung adenocarcinomas (Yamaguchi et al., 2013). These existing examples as well as the suspected role of developmental processes in cancer cell survival are grounds for further investigation into members of this interesting transcription factor family.

Gastric cancer has one of the highest mortality rates of all cancers (Stewart & Wild, 2014). Contributing to this, asymptomatic early stages of the disease mean patients often present with an advanced form of the cancer. Once advanced, gastric cancer is often resistant to treatment and the prognosis is poor. Furthermore, even where early stage treatment has been possible, resurgence frequently occurs. Consequently, there is clear motivation for understanding the molecular mechanisms of disease progression.

The research presented in this issue of *EBioMedicine* by Yoon et al. (2016) furthers our understanding of gastric cancer progression and the role of Nkx factors with their investigation into NKX6, locus 3 (NKX6.3). NKX6.3 is the third member of the Nkx6 subfamily. It is normally expressed in the gastric mucosa and CNS (Alanentalo et al., 2006).

Previous studies by the same authors had found NKX6.3 expression to be reduced in gastric cancers as a result of decreased DNA copy number and allelic loss (Yoon et al., 2015). Building on these findings, the present study correlates reduced NKX6.3 expression with more severe measures of disease progression; higher tumor stage, lymph node metastasis, and TNM (primary **t**umor, regional lymph **n**ode, and **m**etastasis) stage. Mining publically available data from the Gene Expression Omnibus (GEO), demonstrated a strong correlation between reduced expression of NKX6.3 and worse patient outcomes.

In dissecting the molecular basis for these important disease related findings, Yoon et al. demonstrate the role of NKX6.3 in regulating proteins involved in epithelial-mesenchymal transition (EMT). EMT is important in wound healing, organ fibrosis, and — of key relevance here — tumor metastasis. Previous studies implicate EMT in gastric cancer, particularly relating to cell motility and invasiveness (recently reviewed by Huang et al. (2015)). Yoon et al. go on to demonstrate NKX6.3 regulation of the Wnt/β-catenin signalling pathway as the mechanism behind NKX6.3 mediated EMT. Expression profiles of components of the Wnt/β-catenin signalling pathway are shown to be controlled by NKX6.3 and their expression levels perturbed in gastric cancer samples. This finding weaves the present study into a growing body of research linking Wnt/β-catenin signalling to cancer development and progression (Mohammed et al., 2016).

Yoon et al., also show NKX6.3 inhibits Rho-GTPase family proteins which are known to contribute to cancer progression and development. This is important as the Rho-GTPase family proteins RhoA and Rac1 have been shown to be up-regulated in gastric cancers (Pan et al., 2004). This suggests reduced NKX6.3 mediates gastric cancer cell migration and invasion though deregulation of Rho-GTPase family proteins.

Placing this in the context of broader studies, analysis of gastric cancers by The Cancer Genome Atlas Research Network discovered four subtypes; microsatellite unstable tumors, genomically stable tumors, Epstein-Barr virus, and tumors with chromosomal instability (Bass et al., 2014). Interestingly, RHO-family GTPase-activating proteins are implicated in the “genomically stable” tumor subtype. Future studies could further explore whether NKX6.3 expression or biological functions differ in each of these tumor subtypes.

In sum, the studies of Yoon et al. (2016) presented within this issue build on a series of investigations and insights into NKX6.3 and the role of EMT in gastric cancer. Given the prevalence and severity of gastric cancer, the possible utility of NKX6.3 as a biomarker is promising. Additionally, with further investigation there is also the potential to explore treatments targeted at the control of NKX6.3.

## Conflicts of Interest

None.
